# Cyclodextrin Polymer-Embedded NiS/FeS Composite as a Fenton-like Catalyst for the Degradation of Cresol Red

**DOI:** 10.3390/polym17070876

**Published:** 2025-03-25

**Authors:** Eman M. Abd El-Monaem, Jawaher Y. Al Nawah, Mohammed Salah Ayoup, Abdelazeem S. Eltaweil

**Affiliations:** 1 Advanced Technology Innovation, Borg El-Arab, Alexandria, Egypt; emanabdelmonaem5925@yahoo.com; 2 Department of Chemistry, College of Science, King Faisal University, Al-Ahsa 31982, Saudi Arabia; mayoup@kfu.edu.sa; 3 Department of Engineering, Faculty of Technology and Engineering, University of Technology and Applied Sciences, Ibra 400, Oman; 4 Chemistry Department, Faculty of Science, Alexandria University, Alexandria 21523, Egypt

**Keywords:** cresol red, degradation mechanism, metal sulfide, storage–release effect, β-cyclodextrin

## Abstract

Herein, a heterogeneous Fenton-like catalyst was designed by immobilizing iron oxide (FeS) and nickel sulfide (NiS) on the surface of β-cyclodextrin (β-CD), creating a NiS/FeS@β-CD composite for degrading triphenylmethane cresol red dye. Varied instruments were used to study the physical and chemical characteristics of the NiS/FeS@β-CD catalyst. The appropriate catalytic conditions of the Fenton-like degradation of cresol red by NiS/FeS@β-CD were identified, clarifying that the higher degradation % fulfilled 99.86% with an adsorption % of 27.44% at a cresol red concentration = 50 mg/L, NiS/FeS@β-CD dose = 0.01 g, pH = 3, processing temperature = 30 °C, H_2_O_2_ concentration = 100 mg/L, and H_2_O_2_ volume = 1 mL. The kinetic assessments depicted the preference of the second order to represent the Fenton-like degradation of cresol red by NiS/FeS@β-CD. The mechanistic proposition of the adsorption/Fenton-like degradation of cresol red was understood using a quenching test and XPS analysis. Finally, to confirm the durability of NiS/FeS@β-CD, a reusability test was proceeded on the catalyst for five adsorption/Fenton-like degradation runs, with identifying the leaching concentrations of nickel and iron from the catalyst by ICP-OES after each run.

## 1. Introduction

Because of industrial developments, gigantic amounts of polluted substances are released daily into bodies of water, making them unsafe for human use and disrupting aquatic ecosystems. Consequently, environmental researchers have struggled to find an effective remediation strategy for removing such detrimental contaminants from water. Needless to say, many tons of synthetic dyes are used in varied industries like textiles, printing, pharmaceutics, paper, cosmetics, and food [1,2]. This issue results in an elevation in the amounts of dyes released from industries to bodies of water. Meanwhile, the complex aromatic structure and the artificial production of dyes give them high stability and degradation resistance [3]. Triphenylmethane dyes are a sort of persistent synthetic dyes that are applied for dyeing processes, including fat, waxes, leather, varnishes, plastics, and paper. In addition, they are used in textile industries for nylon, wool, silk, and cotton [4]. Cresol red is a triphenylmethane dye that is utilized widely in the textile industry for dyeing polyacrylonitrile-modified nylon, cotton, wool, nylon, and silk [5]. Nevertheless, exposure to cresol red badly affects human health by destroying the kidneys and liver, burning skin, and causing coma, anemia, and irritation [6]. Despite the serious jeopardy of cresol red, the studies that involve removing it from wastewater are still insufficient to understand the dye’s nature and the finest strategy to remove it.

Advanced oxidation processes (AOPs) have gained consideration for their ability to degrade dyes to less- or non-toxic compounds, not just removing them to avoid the formation of noxious byproducts [7]. In addition, AOPs are distinguished by their promising degradation efficacy for dyes, easy operation, and short process time [8]. Amongst AOPs, the Fenton process involves utilizing ferrous-based catalysts for activating oxidants (hydrogen peroxide, H_2_O_2_) and yielding reactive oxygen species (ROS). Meanwhile, pioneering research studies have deduced the promising activity of non-ferrous catalysts, comprising carbon materials, polymers, metal–organic frameworks, MXene, metal sulfides/oxides, etc. [9].

Cyclodextrins (CDs) are cyclic oligosaccharides, constructed from a chain of macro glucopyranose rings in a cone shape [10]. Cyclodextrins are classified as α-CDs, β-CDs, and γ-CDs, based on the number of glucopyranose rings, in which α-CDs, β-CDs, and γ-CDs contain six, seven, and eight glucopyranose rings, respectively [11,12]. Also, cyclodextrins are distinguished according to their cavity diameter, where the cavity sizes of α-CDs, β-CDs, and γ-CDs are 0.47–0.53, 0.6–0.65, and 0.75–0.83 nm [13]. Cyclodextrins have a core–shell morphology, where the core consists of hydrophobic methylene groups, while the shell is constructed of hydrophilic hydroxyl groups [14]. Such unique architecture of the cyclodextrins facilitates the amelioration of their chemical and physical properties by functionalization with advanced substances [15,16]. Furthermore, cyclodextrins possess other propitious advantages, such as safety for humans and animals, cost-effectiveness, availability, and biocompatibility, widening their application scope. It is noteworthy that cyclodextrins have revealed auspicious performance when they are applied as Fenton-like catalysts, since they distinguish themselves with the store–release effect. Therefore, cyclodextrins can store small H_2_O_2_ molecules in their cavity, ease the interaction between H_2_O_2_ and the Fenton catalyst, and release the yielded ^•^OH radicals slowly in a continuous manner [17].

Metal sulfides are substances constructed by bonding earth-rich metal species, such as nickel, iron, tin, and strontium, with sulfur ions with different stoichiometric metal/sulfur ratios [18]. Many fabrication routes have been applied for synthesizing metal sulfides, comprising ball milling, template, thermal decomposition, hydrothermal methods, electrochemical methods, and precipitation [19]. Notably, metal sulfides have exhibited promising results in diverse sectors, like CO_2_ reduction, oxygen generation reactions, lithium-ion batteries, supercapacitors, and catalysis [20,21,22]. Owing to the electric conductivity, electron-rich nature, chemical stability against oxidant attacking, and excellent catalytic activity of metal sulfides, they have been deemed excellent Fenton/Fenton-like catalysts [19,23]. In addition, the sulfur ions in the metal sulfide matrix fasten the transfer of electrons from the metal species to H_2_O_2_, prompting the production rate of the ^•^OH radicals [24,25]. Nonetheless, the Fenton degradation mechanism of the organic pollutants by metal sulfides still needs more investigation, where the studies in this regard are not enough to inspire researchers for further assessments.

Herein, we intended to attract the consideration of researchers to a detrimental dye like cresol red that has no interest, despite its extensive uses in textile industries and its serious risk to human health. Consequently, a Fenton-like catalyst was engineered by immobilizing NiS and FeS over the β-CD surface, producing a NiS/FeS@β-CD composite. Before applying the NiS/FeS@β-CD catalyst, it was analyzed by different characterization instruments to confirm its well-formation and demonstrate its surface charge and morphology, chemical and elemental compositions, and crystalline state. Then, the Fenton-like degradation experiments of cresol red dye by the NiS/FeS@β-CD catalyst were studied, involving identifying the optimal mass ratio between the catalyst components, ideal catalyst dose, suitable H_2_O_2_ concentration, preferable pH and temperature of the catalytic medium, and evaluating the impact of augmenting the MB concentrations. The resultant data from the degradation process of cresol red by NiS/FeS@β-CD were inspected by first-order and second-order models. The mechanistic assumptions of the adsorption/Fenton-like process for degrading cresol red were studied by quenching tests and XPS analysis. The stability of the NiS/FeS@β-CD catalyst was confirmed by performing the recycling test for five adsorption/Fenton-like runs while measuring the metal leaching concentration by ICP-OES after each run.

## 2. Materials and Methods

Nickel acetate (Ni(CH_3_CO_2_)_2_) and trichloromethane (TCM) were purchased from Alpha Chemika (Mumbai, India). Thiourea (SC(NH_2_)_2_) and ferric chloride hexahydrate (FeCl_3_·6H_2_O) were brought from Rankem (Pune, India). Ethylene glycol (EG) and t-butanol (t-BuOH) were provided from Aladdin Reagent Co., Ltd. (Shanghai, China). β-cyclodextrin (β-CD) and cresol red were supplied from MP Biomedicals, LLC (Illkirch-Graffenstaden, France). Ethyl acetate (C_4_H_8_O_2_) and sodium sulfate (Na_2_SO_4_) were provided from Shanghai Chemical Reagent Co., Ltd. (Shanghai, China).

### 2.1. Fabricating Nickel Sulfide

Nickel sulfide was fabricated following the simple hydrothermal route. At beginning, 1.5 g of Ni(CH_3_CO_2_)_2_ was dissolved in 90 mL of distilled H_2_O with potent stirring for 15 min. Then, 3.0 g of SC(NH_2_)_2_ was added to the former solution, followed by stirring for 60 min. The Ni/SC(NH_2_)_2_ mixture was poured in an autoclave and kept in an oven for 10 h at 160 °C. After the autoclave was cooled down, the black NiS powder was separated via centrifugation for 5 min, washed with distilled H_2_O, and heated in an oven at 60 °C for 20 h for drying [1].

### 2.2. Fabricating Iron Sulfide

Iron sulfide was synthesized via the hydrothermal method as follows: At first, 4.05 g of FeCl_3_·6H_2_O and 2.29 g of SC(NH_2_)_2_ were dissolved in an EG/H_2_O mixture with a volume ratio of 20:60. Secondly, the as-prepared reaction solution was transferred in an autoclave and put in an oven for 6 h at 190 °C. Finally, the obtained black solid was collected, washed by distilled H_2_O, and dried at 60 °C for 12 h [2].

### 2.3. Fabricating NiS/FeS@β-CD Composite

**Scheme 1:**
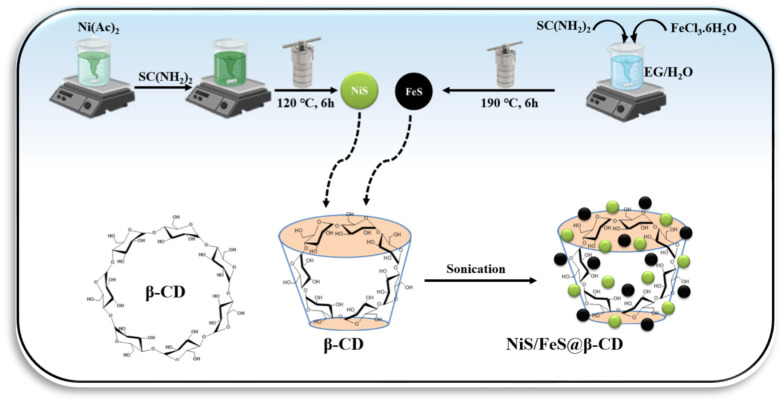
Schematic representation of preparation of NiS/FeS@β-CD composite.

The NiS/FeS@β-CD composites were prepared using the simple post-synthetic procedure. Different stoichiometric weight ratios of NiS, FeS, and β-CD were soaked in 25 mL of distilled water. Three NiS/FeS@β-CD composites were fabricated using weight ratios of NiS, FeS, and β-CD—2:1:1, 1:2:1, and 1:1:2, respectively—and symbolized as 2NiS/FeS@β-CD, NiS/2FeS@β-CD, and NiS/FeS@2β-CD. The composites’ components were kept in their suspensions under sonication for 60 min, and then the composites were collected using the centrifugation instrument and dried in an air oven at 70 °C overnight. A schematic representation for the preparation of the NiS/FeS@β-CD composite is presented in Scheme 1.

### 2.4. Characterization Instruments

The chemical and physical specifications of the NiS/FeS@β-CD composite and its neat material were investigated utilizing Fourier transform infrared (FTIR, Frontier, PerkinElmer), zeta potential (ZP, Malvern), X-ray photoelectron spectroscopy (XPS, ESCALAB 250XI), scanning electron microscopy (SEM, JEOL 7500F), and X-ray diffraction (XRD, PANalytical). The metal leaching proportions from NiS/FeS@β-CD during the Fenton-like degradation of Cresol red was identified by ICP spectroscopy (ICP-OES, Agilent 5110).

### 2.5. Fenton-like Experiments of Degrading the Cresol Red Dye

To study the optimal degradation conditions of cresol red using NiS/FeS@β-CD, a sequence of lab experiments was executed in the adsorption/Fenton-like degradation cycle. Firstly, the adsorption of cresol red by NiS/FeS@β-CD in the absence of H_2_O_2_ was performed for an equilibrium time of 15 min. Secondly, H_2_O_2_ was added to the adsorption cresol red…NiS/FeS@β-CD medium to begin the Fenton-like degradation as follows: (1) A comparison test was conducted between NiS, FeS, β-CD, and the NiS/FeS@β-CD composites to confirm the impact of blinding the neat composites’ component with each other to fabricate efficient composites; in addition, we defined the more efficacious composite between the three NiS/FeS@β-CD composites with different stoichiometric weight ratios. (2) We tested the influence of the pH of the catalytic medium on the degradation % of cresol red by changing the pH range between 3 and 11. (3) We tested the influence of the oxidant concentration, where the concentration of the H_2_O_2_ solution was elevated from 50 to 200 mg/L while recording the degradation % of cresol red. (4) We tested the influence of the oxidant volume, in which the volume of the H_2_O_2_ solution to the cresol red/NiS/FeS@β-CD medium was augmented from 0.5 to 2 mL. (5) We tested the ideal NiS/FeS@β-CD dosage by altering the catalyst dosage from 0.005 to 0.015 g and monitored its effect on the degradation efficiency of cresol red. (6) We tested the thermodynamics of the catalytic medium by raising the temperature of the degradation reaction of cresol red in the H_2_O_2_−NiS/FeS@β-CD system from 30 to 60 °C. (7) We tested the degradation aptitude of NiS/FeS@β-CD at varied cresol red concentrations ranging from 50 to 500 mg/L. The concentrations of initial (C_o_) and undegraded cresol red (C_t_) were identified using a spectrophotometer at a max. wavelength of 456 nm, and the degradation % of cresol red was defined by Equation (1).

(1) $$\mathrm{Degradation}\;\%=\frac{C_{0}-C_{t}}{C_{0}}\times 100$$

### 2.6. Quenching Test

To realize the prevalent ROS, quenchers like t-BuOH and TCM were soaked in the catalytic medium to scavenge ^•^OH and O_2_^•−^, respectively. The test was conducted as follows: 0.01 g of NiS/FeS@β-CD was added into 20 mL of cresol red (50 mg/L) for 15 min, then 1 mL of H_2_O_2_ with a concentration of 100 mg/L and 50 mM of the quenchers was added to the adsorption system while stirring for 30 min. Finally, the controlling ROS was recognized by comparing the degradation % of cresol red in the presence and absence of the quenchers.

### 2.7. Durability Study

To evaluate the durability of the NiS/FeS@β-CD catalyst, reusability and leaching tests were studied for five adsorption/Fenton-like degradation runs of cresol red. The reusability test of NiS/FeS@β-CD was performed as follows: We collected the NiS/FeS@β-CD catalyst from the catalytic medium after the adsorption/Fenton-like degradation run of cresol red and purified it with ethanol. Next, NiS/FeS@β-CD was dried at 70 °C in an oven to reuse it in the following cresol red adsorption/Fenton-like run. Furthermore, the Ni and Fe leaching concentrations were monitored by ICP-OES after each adsorption/Fenton-like run of cresol red.

## 3. Results and Discussion

### 3.1. Characterizing the Chemical/Physical Properties of NiS/FeS@β-CD

#### 3.1.1. FTIR

**Figure 1:**
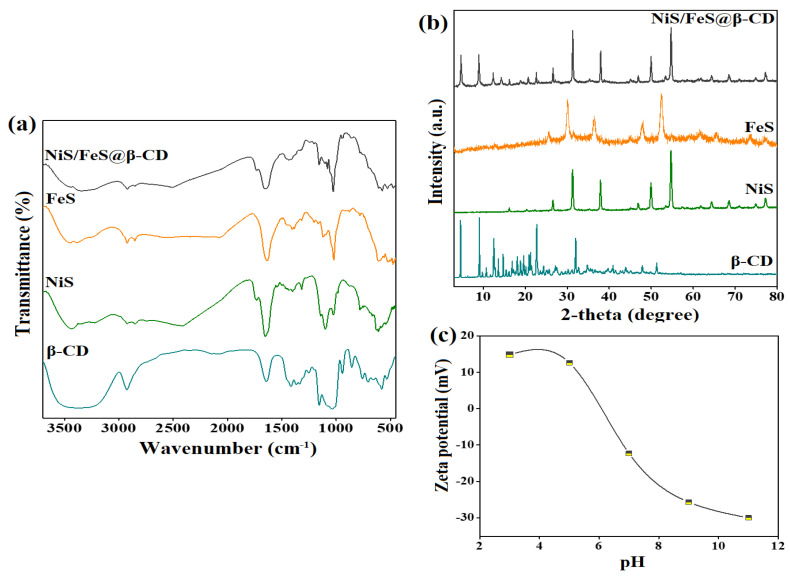
(**a**) FTIR, (**b**) XRD of β−CD, NiS, FeS, and NiS/FeS@β−CD composite, and (**c**) zeta potential of NiS/FeS@β−CD composite.

Figure 1a depicts the chemical structures of β-CD, NiS, FeS, and the NiS/FeS@β-CD composite. The β-CD spectrum manifested the accompanying absorption peaks to the α-1,4-glycosidic bond that connects the glucopyranose units at a wavenumber of 863 cm^−1^ [3]. The bending C−H peaks manifested at 1414, 1337, 1367, and 766 cm^−1^; in addition, the stretching vibrational C−H peak emerged at 2926 cm^−1^. The stretching and vibrating peaks of C−O appeared at the wavenumbers of 1251 and 1153 cm^−1^, sequentially. Furthermore, the related stretching vibrational and vibrating OH peaks were at 3363 and 1642 cm^−1^. The NiS spectrum elucidated the belonging stretching vibrational peaks of Ni−S, where the symmetric bonds manifested at 776 and 630 cm^−1^, and the symmetric bonds appeared at 1143 and 1098 cm^−1^. The corresponding peaks to stretching and bending vibrations of the O−H bond emerged at 3401 and 1648 cm^−1^. In addition, the absorption peak at 1316 cm^−1^ was attributed to the C=S of the SC(NH_2_)_2_ in the preparation of NiS [4,5,6]. The FeS spectrum illustrated the corresponding peaks to C−N of SC(NH_2_)_2_ at wavenumbers of 1454 cm^−1^. The broad absorption peak at 3436 cm^−1^ is assigned to the O−H stretching vibration of the adsorbed H_2_O molecules. The emerged peaks at 615 and 1635 cm^−1^ are ascribed to stretching vibrational S−S bond, and the characteristic absorption peak of S−O appeared at 1118 cm^−1^. The peak with low intensity at 462 cm^−1^ is a fingerprint of Fe−S, and the absorption peak at 1023 cm^−1^ corresponds to Fe−OH [7,8,9]. The NiS/FeS@β-CD spectrum indicated supporting NiS/FeS over the β-CD surface, where the main characteristic peaks of neat NiS, FeS, and β-CD appeared in the FTIR spectrum of the composite.

#### 3.1.2. XRD

The crystallite phases of β-CD, NiS, FeS, and the NiS/FeS@β-CD composite were investigated, as elucidated in the XRD spectra in Figure 1b. The β-CD spectrum depicted its typical diffraction peaks at 2θ = 8.81°, 10.68°, 11.68°, 12.46°, 14.70°, 17.16°, 21.18°, 22.68°, and 32.66° [10,11]. The NiS spectrum clarified the distinguishing peaks at 2θ = 16.13°, 31.24°, 37.88°, 46.87°, 49.89°, 54.68°, 64.33°, and 68.44° coincide with the planes of (110), (101), (220), (102), (131), (401), (241), and (600) [12,13,14]. The FeS spectrum implied the distinctive peaks at 2θ = 25.58°, 30.05°, 36.41°, 44.91°, 47.89°, 52.40°, 61.77°, 65.35°, 73.57°, and 77.01° accompanied the planes of (220), (311), (400), (422), (511), (440), (533), (444), (731), and (800) [15]. The NiS/FeS@β-CD spectrum demonstrated the diffraction peaks of NiS, FeS, and β-CD, denoting the effective combination of these neat components to fabricate the composite.

#### 3.1.3. Zeta Potential

Since adsorption is the initial stage of the catalysis reaction, it was fundamental to determine the surface charge over the NiS/FeS@β-CD surface, as demonstrated in Figure 1c. The zeta potential results implied that the zero-charge point of NiS/FeS@β-CD is at pH = 6.08, meaning it charges the NiS/FeS@β-CD catalyst with negative charges at pH lower than 6.08, in which the measured zeta potential values at pH = 3 and 5 were 14.88 and 12.63 eV. Consequently, the NiS/FeS@β-CD surface would be available to adsorb anionic pollutants like cresol red in acidic media via electrostatic interactions. On the contrary, the surface charge of NiS/FeS@β-CD turned negative when the pH exceeded 6.08, denoting the suitability of the catalyst to adsorb cationic pollutants in alkaline and neutral media.

#### 3.1.4. SEM

**Figure 2:**
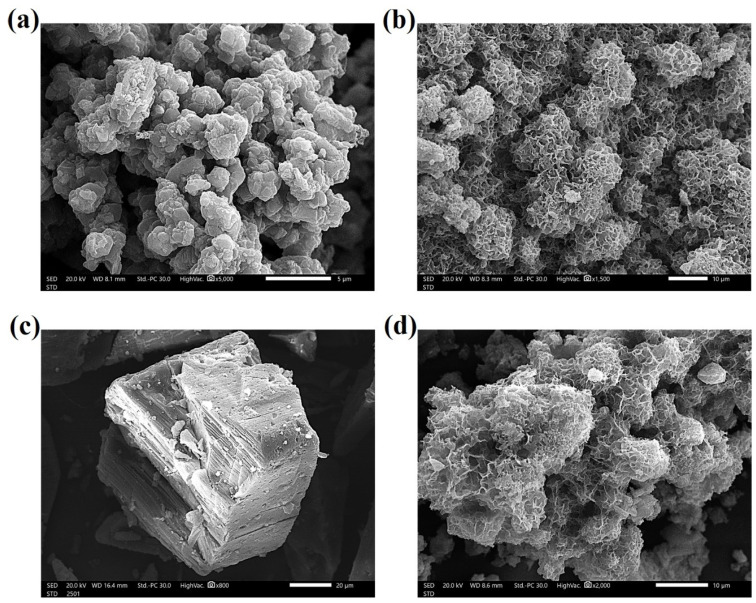
The SEM images of (**a**) NiS, (**b**) FeS, (**c**) β−CD, and (**d**) the NiS/FeS@β−CD composite.

The morphologies of β-CD, NiS, FeS, and the NiS/FeS@β-CD composite were studied by SEM, as shown in Figure 2a–d. The morphology of the NiS particles was irregular and aggregated in the form of interconnected clusters. The morphology of the particles of FeS looks like a dandelion flower shape. The morphology of β-CD appeared as exfoliated sheets sticking together. The sheet of β-CD was rough with wide holes, suggesting the suitability of its surface to be a platform to support the particles of NiS and FeS. Furthermore, these cavities of β-CD could be beneficial in adsorbing cresol red molecules by a pore-filling mechanism and the storage–release of the H_2_O_2_ molecules during the Fenton-like reaction, providing high concentrations of ^•^OH. The SEM image of the NiS/FeS@β-CD composite revealed the growth of the particles of FeS and NiS on the β-CD sheets.

#### 3.1.5. XPS

**Figure 3:**
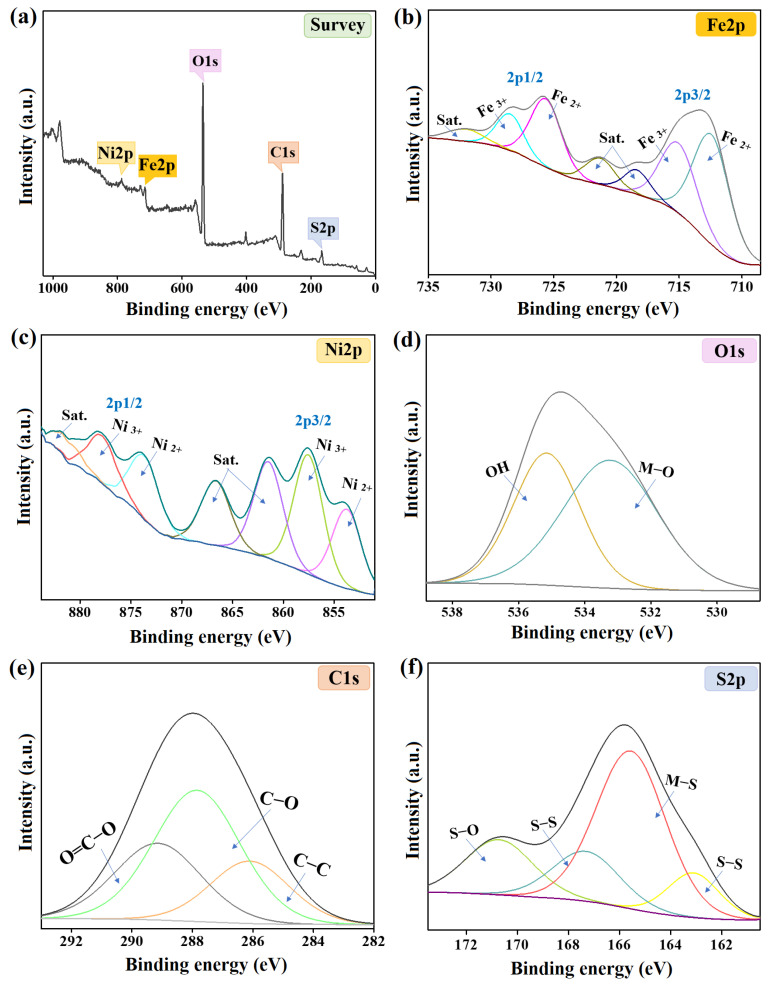
The XPS spectra of the NiS/FeS@β−CD catalyst; (**a**) Wide-spectrum, (**b**) Fe2p, (**c**) Ni2p, (**d**) O1s, (**e**) C1s, and (**f**) S2p.

The elemental composition of the NiS/FeS@β-CD catalyst was investigated by XPS, as revealed in Figure 3a–f. The wide spectrum of this/FeS@β-CD composite showed the distinguishing peaks of oxygen, carbon, iron, sulfur, and nickel at 534.87, 288.38, 714.39, 166.29, and 854.08%, respectively. The iron spectrum illustrated the Fe^3+^ peaks of 2p3/2 and 2p1/2 at 714.69 and 727.22 eV with an atomic % of 28.98%; furthermore, the Fe^2+^ peaks of 2p3/2 and 2p1/2 manifested at 712.28 and 724.81 eV with an atomic % of 39.97%. The nickel pattern elucidated the characteristic peaks of Ni^2+^ of 2p3/2 and 2p1/2 at 853.77 and 873.66 eV, respectively, with a total atomic percent of 29.10%. In addition, the related peaks of Ni^3+^ 2p3/2 and 2p1/2 appeared at 857.54 and 877.80 eV, and the atomic % of Ni^3+^ in the NiS/FeS@β-CD composite was 33.96% [16]. The oxygen spectrum clarified the belonging XPS peaks of Ni/Fe−O and O−H at 533.23 and 535.15 eV with atomic percentages of 57.81 and 42.19%, sequentially. The carbon spectrum demonstrated the corresponding peaks to the carbon-containing groups in the NiS/FeS@β-CD composite at 286.10, 289.14, and 287.85 eV, which are ascribed to C−C, O=C−O, and C−O with atomic percentages of 21.60, 28.95, and 49.45%, respectively. The sulfur spectrum of the NiS/FeS@β-CD composite clarified the two peaks of S−S at 163.11 and 167.31 eV with an atomic % of 27.79% [17]. While the emerged XPS peaks at 170.72 and 165.56 eV were accompanied by S−O and Fe/Ni−S, and their atomic % were 18.42 and 53.80% [18].

### 3.2. Discussing the Results of the Lab Experiments of Degrading Cresol Red

#### 3.2.1. Investigating the Best Stoichiometric Ratio

**Figure 4:**
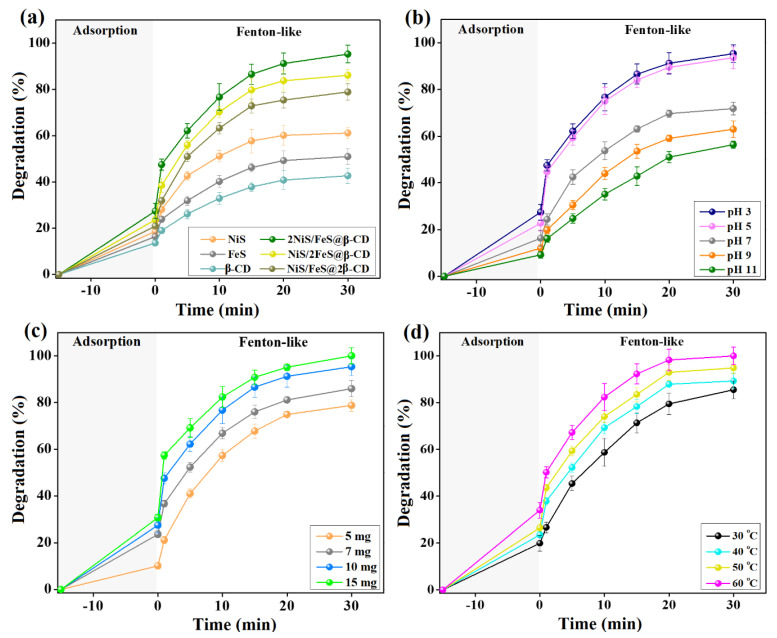
(**a**) Comparison test between the degradation efficacy of the 2NiS/FeS@β−CD composites and their neat materials toward cresol red. (**b**) Investigating the appropriate pH. (**c**) Determining the ideal dose of 2NiS/FeS@β−CD. (**d**) Assessing the influence of the processing temperature of the degradation % of cresol red.

Figure 4a represents the experimental observations from the comparison test between the efficacy of NiS, FeS, β-CD, 2NiS/FeS@β-CD, NiS/2FeS@β-CD, and NiS/FeS@2β-CD to degrade cresol red. It was monitored that all the examined samples have inferior adsorption aptitude towards cresol red, where the adsorption % of NiS, FeS, β-CD, 2NiS/FeS@β-CD, NiS/2FeS@β-CD, and NiS/FeS@2β-CD was 18.63, 16.40, 13.63, 27.44, 23.68, and 20.96%, respectively. Meanwhile, the catalytic activity of NiS, FeS, β-CD, 2NiS/FeS@β-CD, NiS/2FeS@β-CD, and NiS/FeS@2β-CD towards the degradation of cresol red was 61.16, 51.00, 42.69, 95.32, 86.15, and 78.92%, respectively. The drastic improvements in the Fenton-like degradation % of cresol red by the NiS/FeS@β-CD composites compared to the pristine substances were because of the combination between the active metals (viz., Ni^2+^ and Fe^2+^). In addition, the storage–release effect of β-CD promotes the production of the ^•^OH radicals. Also, unsaturated sulfur can enhance the proportion of yielded ^•^OH radicals since it ameliorates transferring electrons from the near metal species to H_2_O_2_ [19].

#### 3.2.2. Investigating the Optimal pH

The pH influence on the Fenton-like degradation of cresol red by the 2NiS/FeS@β-CD catalyst was studied at a pH scale of 3–11, as shown in Figure 4b. The lab experiments demonstrated a decline in the adsorption percent of cresol red from 27.44 to 9.19% and Fenton-like degradation percent from 95.32 to 56.38% by elevating the catalytic pH media from 3 to 11. This diminution in the efficiency of 2NiS/FeS@β-CD towards the adsorption of cresol red when increasing pH over 3 can be ascribed to the decline in the positive charges on the composite’s surface, decreasing its electrostatic interactions with the anionic cresol red [20]. Furthermore, the suitability of the highly acidic medium to degrade cresol red in the H_2_O_2_−2NiS/FeS@β-CD medium can be assigned to the possible formation of hydro per-oxy ions by the interactions between H_2_O_2_ and the ample ^−^OH ions in the alkaline media, as demonstrated in Equation (2). It is well known that hydro per-oxy ions possess a potent affinity toward metal species in catalytic media (Equation (3)) [21]. In addition, the self-degradation of H_2_O_2_ to oxygen and water in alkaline media is elucidated in Equation (4) [22].

H_2_O_2_ + OH^−^ → OOH^−^ + H_2_O (2)

M + OOH^−^ → M…^•^OOH (3)

2H_2_O_2_ → O_2_ + 2H_2_O (4)

#### 3.2.3. Investigating the Ideal Catalyst Dosage

The results from testing the adsorption/Fenton-like degradation efficiency of cresol red by different doses of the 2NiS/FeS@β-CD composite are illustrated in Figure 4c. The adsorption % of cresol red was 10.10 % when the 2NiS/FeS@β-CD dosage was 0.005 g, while it attained 30.67 by elevating the composite amount to 0.015 g. These observations can be ascribed to the enrichment in the adsorption sites by augmenting the proportion of 2NiS/FeS@β-CD. It was monitored that the adsorption % of cresol red almost doubled when the 2NiS/FeS@β-CD dosage was raised from 5 to 10 mg. In comparison, the further increase in the dosage to 15 mg revealed an inconsiderable effect on the adsorption % of cresol red. This finding suggested that the adsorption reaction of cresol red (50 mg/L) reached an equilibrium state with 10 mg of 2NiS/FeS@β-CD [23]. The over-increase in the 2NiS/FeS@β-CD dose causes a cohesive interaction between the catalyst’s particles, resulting in aggregations, a decline in the available surface area per unit weight of 2NiS/FeS@β-CD and an increment in the diffusion path length [24]. Likewise, the increase in the active sites that enhance ^•^OH production by increasing the 2NiS/FeS@β-CD proportion to 0.01 g results in a magical amelioration in the Fenton-like degradation % of cresol red, which reached 95.32 % [25]. So, 10 mg of 2NiS/FeS@β-CD could provide sufficient ^•^OH radicals to efficiently degrade 50 mg/L of cresol red. Meanwhile, the further elevation in the dosage of the Fenton-like catalyst showed an increase in the degradation % by less than 5 %, which was an inferior impact compared to the economic cost.

#### 3.2.4. Investigating the Influence of Processing Temperature

The processing temperature of the Fenton-like degradation process of cresol red by 2NiS/FeS@β-CD was elevated from room temperature to 60 °C to understand the thermodynamics of the adsorption/catalytic medium, as illustrated in Figure 4d. The adsorption % of cresol red enhanced from 19.90 to 33.95% by raising the processing temperature. This observation can be anticipated by augmenting the Brownian motion of the cresol red molecules, prompting their adsorption onto the 2NiS/FeS@β-CD surface. Similarly, the catalytic degradation % of cresol red increased from 85.51 to 100% since the higher temperature facilitates the diffusion of H_2_O_2_ and cresol red to the catalyst surface, strengthens the interaction between H_2_O_2_ and 2NiS/FeS@β-CD, and fosters yielding ^•^OH radicals [26].

#### 3.2.5. Investigating the Optimal H_2_O_2_ Concentration/Volume

**Figure 5:**
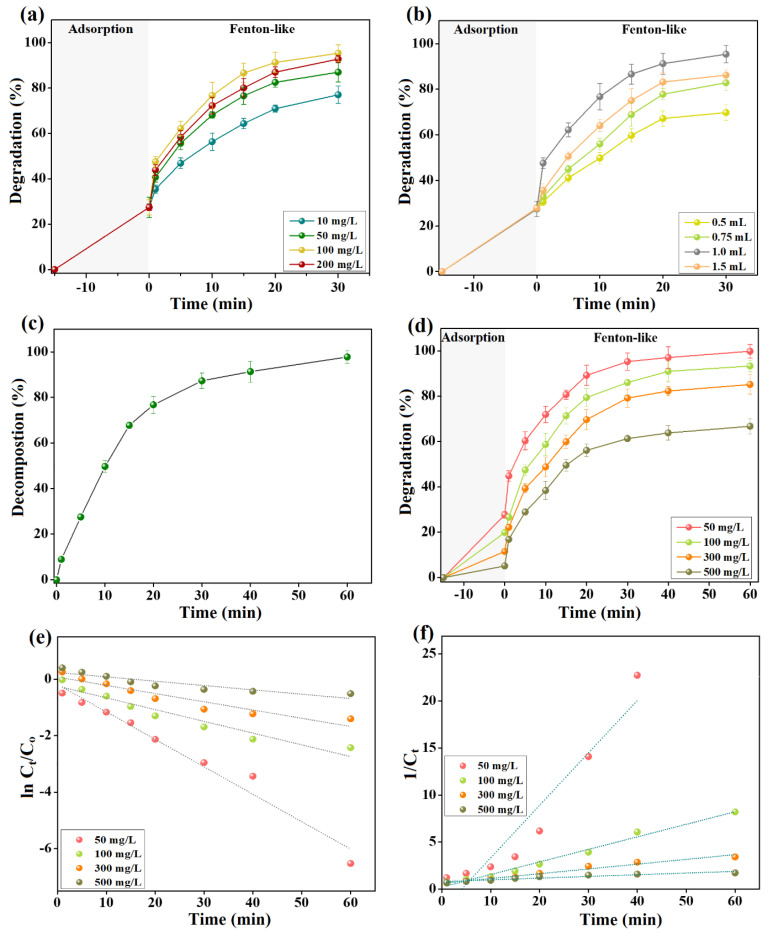
(**a**) Optimizing the best concentration of H_2_O_2_. (**b**) Identifying the appropriate volume of H_2_O_2_. (**c**) Determining the equilibrium decomposition time of H_2_O_2_. (**d**) Defining the catalytic activity of 2NiS/FeS@β−CD toward the varied cresol red concentrations. Analyzing the obtained data by kinetic models: (**e**) first order and (**f**) second order.

Figure 5a,b depict the experimental findings of inspecting the capability of the Fenton-like degradation of cresol red by 2NiS/FeS@β-CD in the presence of varied H_2_O_2_ concentrations and volumes. The increment in the H_2_O_2_ concentration from 10 to 100 mg/L improved the degradation efficacy of cresol red from 77.02 to 95.32%. Nevertheless, the over-raising in the H_2_O_2_ concentration to 200 mg/L slightly diminished the efficiency of cresol red degradation to 92.73%. Furthermore, a drastic increase in the Fenton-like degradation % of cresol red from 69.75 to 95.32% when the volume of H_2_O_2_ with a concentration of 100 mg/L was doubled from 0.5 to 1.0 mL. In addition, the increase in the added H_2_O_2_ volume to 1.5 mL declined the cresol red degradation % to 86.19%. The perceptible improvement in the degradation % of cresol red by elevating the concentration and volume of H_2_O_2_ to 100 mg/L and 1 mL, respectively, is most likely due to the enhancement in the yielding amounts of ^•^OH radicals. However, the over-concentration and volume of H_2_O_2_ can attack the existing ^•^OH radicals in the catalytic medium, as demonstrated in Equations (5) and (6).

H_2_O_2_ + ^•^OH → HOO^•^ + H_2_O (5)

HOO^•^ + ^•^OH → H_2_O + O_2_ (6)

#### 3.2.6. Investigating the Equilibrium Decomposition Time of H_2_O_2_

Figure 5c clarified the decomposition of H_2_O_2_ using 2NiS/FeS@β-CD for an hour in the absence of the cresol red dye. It was recorded that the decomposition of the H_2_O_2_ molecules attained equilibrium after half an hour and reached the maximum decomposition % of 97.78% after an hour. This finding suggested that the degradation percent of cresol red by 2NiS/FeS@β-CD would reach its maximum value after a reaction time of 60 min since H_2_O_2_ would almost decompose completely.

### 3.3. Kinetic Investigations

The catalytic activity of the Fenton-like 2NiS/FeS@β-CD catalyst towards high and low cresol red concentrations ranging from 50 to 500 mg/L was studied, as revealed in Figure 5d. The lab experiments showed that the adsorption percentages of cresol red by 2NiS/FeS@β-CD were 27.66, 19.90, 11.54, and 5.15% when the cresol red concentrations were 50, 100, 300, and 500 mg/L. This obvious decline in cresol red adsorption % with elevating concentrations is because of the fewer active binding sites on the 2NiS/FeS@β-CD surface compared to the amounts of the cresol red molecules at high concentrations. Furthermore, the Fenton-like degradation of cresol red declined from 99.87 to 93.23, 85.17, and 66.72% by increasing the cresol red concentrations from 50 to 100, 300, and 500 mg/L because of the insufficient amounts of yielding ^•^OH radicals in the catalytic medium for degrading such high amounts of cresol red molecules.

The former experimental results were inspected by first-order and second-order models (Equations (7) and (8)) to understand the kinetics of the Fenton-like degradation of cresol red by 2NiS/FeS@β-CD. Figure 5e,f illustrate the first-order and second-order curves, and the derived parameters from them are listed in Table 1. The kinetic investigations depicted the appropriateness of the second order to model the Fenton-like degradation process of cresol red by 2NiS/FeS@β-CD, where its R^2^ values are higher than those of the first order. In addition, the kinetic rate constants of 2NiS/FeS@β-CD toward cresol red with concentrations of 50, 100, 300, and 500 mg/L were 0.5579, 0.1335, 0.0508, and 0.0181 min^−1^. Obviously, the rate constant of the cresol red degradation reaction declined with increasing initial concentrations of the dye because of the complex balancing act of some presented factors in the catalytic medium, including ^•^OH production/consumption and the possible competitive reaction of cresol red molecules with their degradation intermediates [27,28,29].

(7) $$ln\;\frac{C_{t}}{C_{o}}=-k_{1}t$$

(8) $$\frac{1}{C_{t}}=\frac{1}{C_{o}}+k_{2}t$$

*k*_1_ and *k*_2_ represent the first-order and second-order rate constants.

### 3.4. Quenching Test

**Figure 6:**
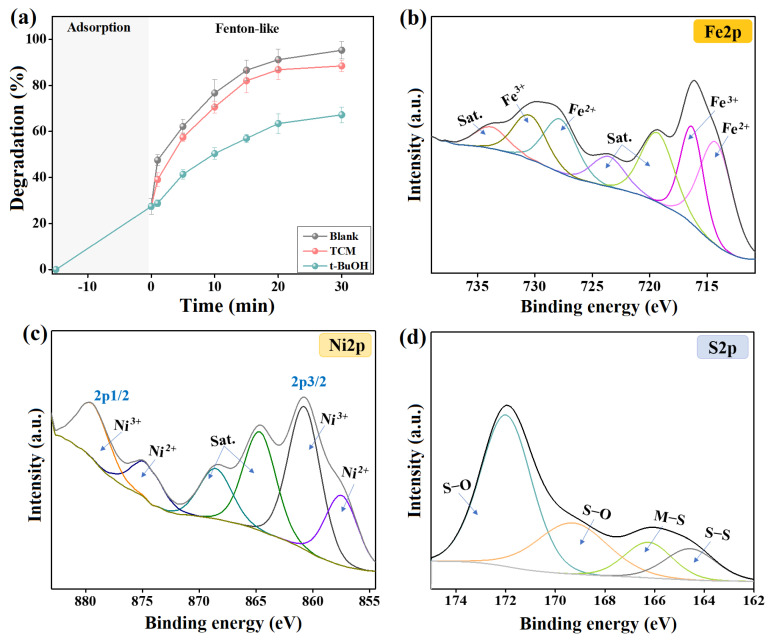
(**a**) Quenching test of the catalytic degradation of 2NiS/FeS@β−CD toward cresol red and the XPS spectra of the used 2NiS/FeS@β−CD, (**b**) Fe 2p, (**c**) Ni 2p, and (**d**) S 2p.

Indeed, ^•^OH and O_2_^•−^ are the ROS in Fenton-like catalytic media that attack organic pollutants and degrade them to lower- or non-toxic smaller molecules. Consequently, highly reactive quenchers against ^•^OH and O_2_^•−^ were added separately to the Fenton-like degradation medium of cresol red, which were TCM and t-BuOH to retard O_2_^•−^ and ^•^OH, respectively [30]. The quenching test (Figure 6a) demonstrated a drastic decline in the efficiency of cresol red degradation by 28.08% in the presence of t-BuOH. In comparison, TCM only diminished the degradation % of cresol red by 6.82%. This finding indicated that ^•^OH is the active ROS in the degradation reaction of cresol red by 2NiS/FeS@β-CD and O_2_^•−^ did not participate in the Fenton-like reaction [31]. In addition, the Fenton-like degradation of cresol red proceeded throughout the catalytic radical pathway, not the oxygen vacancy mechanism.

### 3.5. Degradation Mechanism

The experimental work proceeded in an adsorption/Fenton-like run to examine the participation of the adsorption in the degradation efficiency of cresol red by 2NiS/FeS@β-CD. Based on the experimental results, the adsorption exhibited a low contribution in the degradation process of cresol red with a higher adsorption % of 27.65 when the cresol red concentration was 50 mg/L. The adsorption of cresol red onto the 2NiS/FeS@β-CD surface may occur as an initial step.

(1) Electrostatic interaction is the most prevailing adsorption pathway in almost all adsorption processes, while the surface of the 2NiS/FeS@β-CD catalyst carries a low positive charge amount on its surface at pH = 3. So, the electrostatic interaction between the lowly positively charged 2NiS/FeS@β-CD and the anionic cresol red is not quite potent. This may be the cause of the low adsorption efficacy of 2NiS/FeS@β-CD toward cresol red molecules.

(2) Coordinate bonds are one more adsorption pathway that is considered in removing cresol red, owing to the presence of hydroxyl and sulfite groups. Meanwhile, the 2NiS/FeS@β-CD composite contains unsaturated metal ions, comprising nickel and iron that can bond to the hydroxyl and sulfite groups of cresol red [32].

(3) n-π interaction is an effective pathway, contributing to the adsorption of the organic pollutants that contain π-orbitals in their aromatic rings [33]. Cresol red molecules contain π-orbitals that could accept the transferred electrons from the oxygenating functional groups of 2NiS/FeS@β-CD. Thereby, n-π interaction could participate in the adsorption process of cresol red onto the surface of 2NiS/FeS@β-CD.

Then, in the second step, the Fenton-like degradation of cresol red by 2NiS/FeS@β-CD was supposed to occur as follows:

(1) ^•^OH is produced by the storage–release effect of β-CD, in which it possesses individual merit in catching the small molecules of H_2_O_2_ from the catalytic medium and storing them in its wide cavities. As a result, β-CD enhances the production of ^•^OH and facilitates its continuous and gradual release. This property of β-CD addresses a significant issue with H_2_O_2_, which is its tendency for self-decomposition, as it reduces the concentration of H_2_O_2_ molecules in water [34].

(2) H_2_O_2_ is activated by the Fe species-containing 2NiS/FeS@β-CD to produce ^•^OH radicals. By comparing the XPS spectra of the Fe species before and after the Fenton-like degradation of cresol red (Figure 6b), we noticed a change in the peak positions with a decline in the atomic Fe^2+^/Fe^3+^ ratio from 1.379 to 1.108. This observation could be explained by the consumption of Fe^2+^ in the activation of H_2_O_2_ and the creation of ^•^OH radicals, as clarified in Equation (9). In addition, Fe^2+^ ions can also participate in recovering Ni^3+^, as elucidated in Equation (10), since the redox potentials of Fe^2+^/Fe^3+^ and Ni^2+^/Ni^3+^ are 0.77 and 2.94 V, respectively.

Fe^2+^ + H_2_O_2_ → Fe^3+^ + ^•^OH + OH^−^ (E^⁰^ of Fe^2+^/Fe^3+^ = 0.77 V) (9)

Fe^2+^ + Ni^3+^ → Fe^3+^ + Ni^2+^ (10)

(3) H_2_O_2_ is activated by the Ni ions in the 2NiS/FeS@β-CD catalyst, as elucidated in Equation (11), to yield active ^•^OH radicals. The Ni spectra of neat/used 2NiS/FeS@β-CD catalysts manifested shifting in the Ni^2+^ and Ni^3+^ of 2p3/2 and 2p1/2, as shown in Figure 6c. Furthermore, the atomic percent of the Ni^2+^/Ni^3+^ in the used 2NiS/FeS@β-CD catalyst (0.856) was lower than that of the neat catalyst (0.778).

Ni^2+^ + H_2_O_2_ → Ni^3+^ + ^•^OH + OH^−^ (Ni^2+^/Ni^3+^ = 2.94 V) (11)

**Figure 7:**
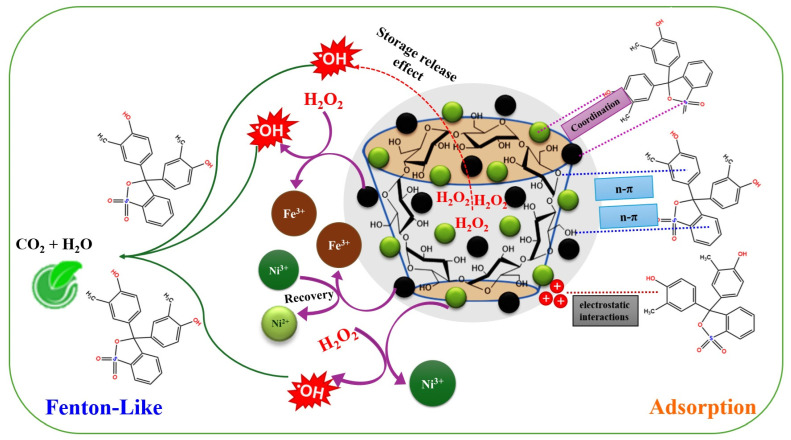
The adsorption/Fenton-like mechanism of degrading cresol red by the 2NiS/FeS@β−CD catalyst.

(4) The activity of the Ni and Fe species is ameliorated by bonded unsaturated sulfur, since it prompts transferring electrons from the metal species through the metal–sulfur pathway. Hence, the presence of unsaturated sulfur in the 2NiS/FeS@β-CD catalyst strengthens the concentration of the produced ^•^OH radicals. The sulfur spectrum of the used 2NiS/FeS@β-CD catalyst (Figure 6d) observed shifting in the peaks of the sulfur-containing functional groups, compared to the peaks of the pure catalyst, reflecting the contribution of the sulfur ions in activating H_2_O_2_. Figure 7 clarifies the adsorption/Fenton-like mechanism of degrading cresol red by the2NiS/FeS@β-CD catalyst.

### 3.6. Proposed Pathway for Cresol Red Degradation

**Figure 8:**
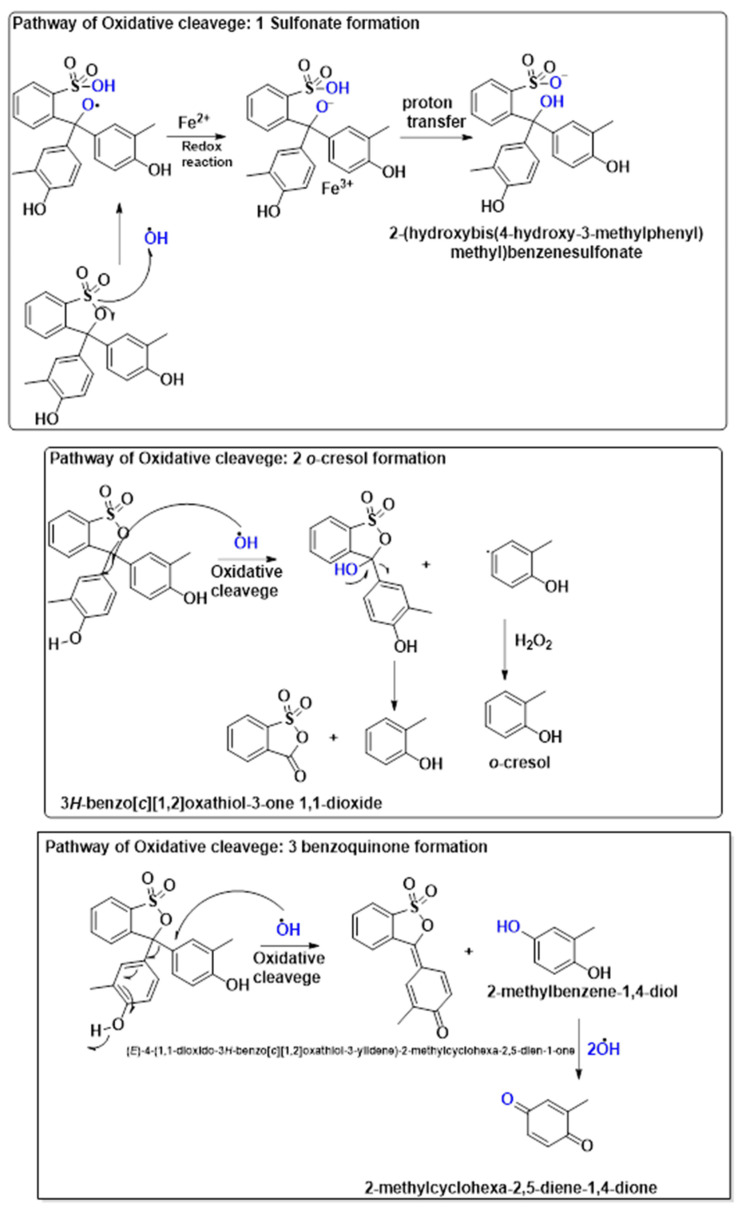
The proposed pathway for cresol red degradation by the Fenton-like reaction using the 2NiS/FeS@β−CD catalyst.

The oxidative degradation of cresol red could proceed through two key intermediate pathways: sulfonate formation and benzoquinone formation. In the sulfonate pathway, redox reactions involving M^2+^/M^3+^ facilitate electron transfer, leading to proton transfer and the formation of 2-(hydroxybis(4-hydroxy-3-methylphenyl)methyl)benzenesulfonate, which enhances water solubility for further degradation. Alternatively, the benzoquinone pathway involves hydroxyl radical-induced cleavage, yielding 2-methylbenzene-1,4-diol, which is further oxidized to 2-methylcyclohexa-2,5-diene-1,4-dione. These pathways play a crucial role in breaking down cresol red into smaller, more biodegradable compounds, aiding in environmental detoxification and wastewater treatment. The proposed pathway for the degradation of cresol red by the 2NiS/FeS@β-CD catalyst is presented in Figure 8. The suggested pathways of degradation were classified into three mechanistic schemes: the oxidative cleavage pathway, directed to both sulfonate formation and o-cresol formation, where the hydroxyl free radical plays a key role in destroying cresol red by attacking it in different centers, which finally gives the small species of o-cresol, where the GC-MS result (Figure S1) reported a strong signal with a retention time of 26.8 min, which nearly aligned with the Rt that has been reported in the literature [35] at 27.2 min; the third pathway revealed the formation of 2-methyl-1,4-benzoquinone. It is worthy to mention that the most of the resultant degradation has no reported retention time in the literature, where the GC-MS spectrum reported plenty of signals with different retention times, which may be aligned with the formed species.

### 3.7. Durability Study

**Figure 9:**
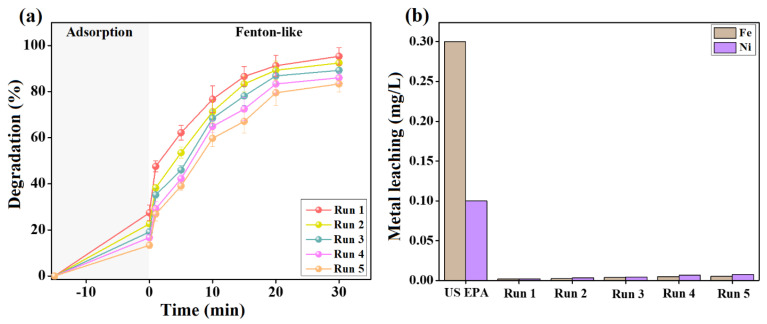
(**a**) Reusability test of the 2NiS/FeS@β−CD catalyst for five adsorption/Fenton-like degradation runs of cresol red. (**b**) Leaching test of the 2NiS/FeS@β−CD catalyst after each adsorption/Fenton-like degradation run of cresol red.

After confirming the excellent catalytic activity of the Fenton-like 2NiS/FeS@β-CD catalyst towards the degradation of cresol red, it was crucial to ensure its durability throughout the reusability and metal leaching tests. The reusability study of 2NiS/FeS@β-CD was performed for five adsorption/Fenton-like runs, as illustrated in Figure 9a, clarifying slight diminutions in the efficiency of both the adsorption and Fenton-like degradation of cresol red. The experimental observations denoted a decline in the adsorption % of cresol red after the first run from 27.44 to 22.64, 19.12, 16.69, and 13.34% after the second, third, fourth, and fifth runs; in addition, the Fenton-like efficiency of degrading cresol red decreased from 95.32 to 92.43, 89.19, 86.02, and 83.30, respectively. These results are probably due to the weight loss of the 2NiS/FeS@β-CD catalyst through its separation and regeneration stages.

Furthermore, metal leaching from the 2NiS/FeS@β-CD catalyst after five adsorption/Fenton-like runs of cresol red was examined. As depicted in Figure 9b, the nickel and iron leaching percentages from 2NiS/FeS@β-CD attained 0.0075 and 0.0054 mg/L after the fifth adsorption/Fenton-like degradation run of cresol red. Meanwhile, the US EPA has administrated that the highest acceptable concentrations of nickel and iron in water are 0.1 and 0.3 mg/L. Consequently, 2NiS/FeS@β-CD is a stable catalyst with non-toxicity merit, in which the low leached nickel and iron concentrations would not cause secondary water contaminants.

## 4. Conclusions

The designed Fenton-like 2NiS/FeS@β-CD catalyst exhibited a promising efficacy towards degrading cresol red, where the higher degradation % reached 99.86% with an adsorption % of 27.44% at a cresol red concentration = 50 mg/L, H_2_O_2_ volume = 1 mL, processing temperature = 30 °C, NiS/FeS@β-CD dose = 0.01 g, pH = 3, and H_2_O_2_ concentration = 100 mg/L. The kinetic investigations demonstrated the suitability of the second-order model to model the degradation of cresol red by NiS/FeS@β-CD. The quenching test clarified that the Fenton-like degradation of cresol red by NiS/FeS@β-CD proceeds via the radical pathway with the controlling of the ^•^OH radicals in the degradation reaction. The reusability test of the NiS/FeS@β-CD catalyst elucidated a decline in the adsorption % and Fenton-like degradation % from 27.44 and 95.32% to 13.34 and 83.30%. In addition, the leached nickel and iron from NiS/FeS@β-CD reached 0.0075 and 0.0054 mg/L after the fifth adsorption/Fenton-like degradation run of cresol red. The mechanistic assumption suggested the adsorption of cresol red onto the NiS/FeS@β-CD surface occurs via electrostatic interaction, coordination bonds, and n-π interactions. Furthermore, the Fenton-like degradation of cresol red proceeded by forming a continuous redox cycle between nickel and iron species. In addition, the storage–release merit of β-CD boosted the production of •OH and controlled releasing the radicals to the catalytic medium. Also, the sulfur species ameliorated yielding •OH, where they facilitated transferring the electrons from the near metals to the catalytic medium. According to our study, the designed NiS/FeS@β-CD catalyst revealed efficacious catalytic performance, high stability, and excellent reusability during the degradation of cresol red. Nevertheless, NiS/FeS@β-CD has a limitation: the weight loss during its separation takes a long time with weight loss. This problem is most likely the main reason for the cycle-by-cycle decline in the catalytic activity of NiS/FeS@β-CD towards the cresol red dye. Therefore, we recommend decorating the NiS/FeS@β-CD catalyst with a magnetic substance to facilitate its separation via a magnet. Also, the incorporation of NiS/FeS@β-CD into beads could be another possible solution to provide perfect and fast separation from the catalyst.

## Figures and Tables

**Table 1:** Kinetic parameters of the Fenton-like degradation process of cresol red by the 2NiS/FeS@β-CD catalyst.

Kinetic Model	CR Concentrations (mg/L)			
	50	100	200	300
First order				
*k*_1_	0.0972	0.0416	0.0291	0.0153
R^2^	0.972	0.937	0.904	0.833
Second order				
*k*_2_	0.5579	0.1335	0.0508	0.0181
R^2^	0.917	0.985	0.969	0.899

## Data Availability

The original contributions presented in this study are included in the article and Supplementary Material. Further inquiries can be directed to the corresponding authors.

## Supplementary Materials

The following supporting information can be downloaded at: https://www.mdpi.com/article/10.3390/polym17070876/s1, Figure S1: GC-MS of the degraded cresol red by the Fenton-like 2NiS/FeS@β-CD catalyst.
