# Supplementary material for: Cyclodextrin Polymer-Embedded NiS/FeS Composite as a Fenton-like Catalyst for the Degradation of Cresol Red
Source: Polymers (Basel). 2025 Mar 25;17(7):876. doi: 10.3390/polym17070876 (PMC11991624; doi:10.3390/polym17070876)
Supplement: Supplementary file 1 [file polymers-17-00876-s001.zip › polymers-3494003-supplementary.pdf]

polymers

Supplementary

Cyclodextrin Polymer-Embedded NiS/FeS Composite as a Fenton-like Catalyst for the Degradation of Cresol Red

Eman M. Abd El-Monaem [1], Jawaher Y. Al Nawah [2,*], Mohammed Salah Ayoup [2] and Abdelazeem S. Eltaweil [3,4,*]

[1] Advanced Technology Innovation, Borg El-Arab, Alexandria, Egypt; emanabdelmonaem5925@yahoo.com
[2] Department of Chemistry, College of Science, King Faisal University, Al-Ahsa 31982, Saudi Arabia; mayoup@kfu.edu.sa
[3] Department of Engineering, Faculty of Technology and Engineering, University of Technology and Applied Sciences, Ibra 400, Sultanate of Oman
[4] Chemistry Department, Faculty of Science, Alexandria University, Alexandria 21523, Egypt
* Correspondence: jalnawah@kfu.edu.sa (J.Y.A.N.); abdelazeemeltaweil@alexu.edu.eg (A.S.E.)

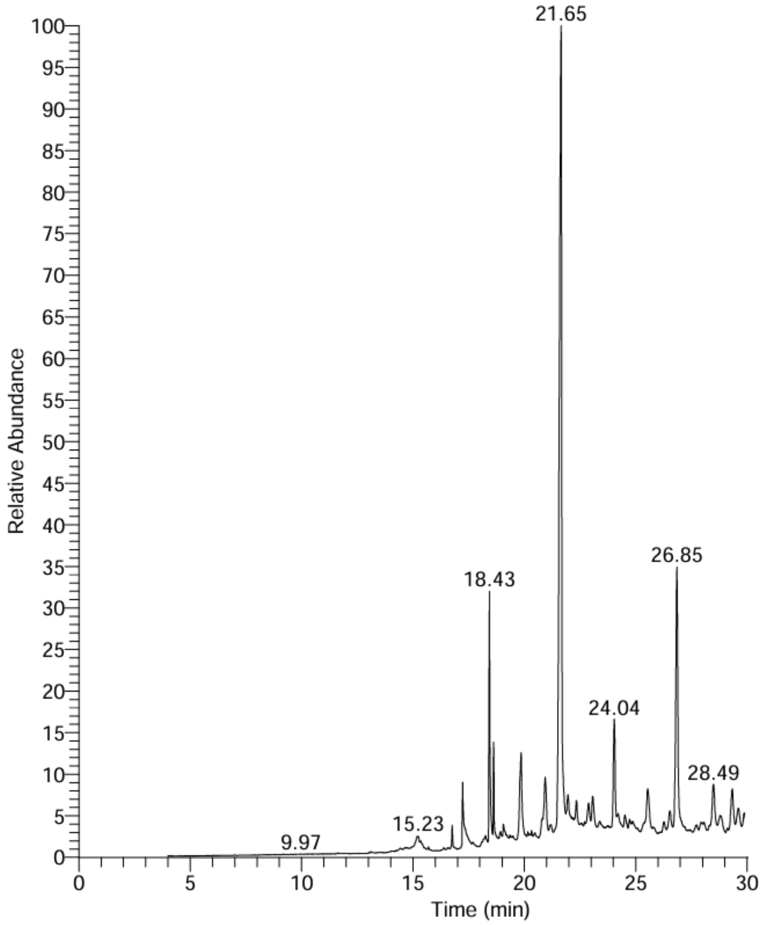

Figure S1. GC-MS of the degraded cresol red by the Fenton-like 2NiS/FeS@β-CD catalyst.
